# Proteomic and Metabolomic Signatures Associated With the Immune Response in Healthy Individuals Immunized With an Inactivated SARS-CoV-2 Vaccine

**DOI:** 10.3389/fimmu.2022.848961

**Published:** 2022-05-24

**Authors:** Yi Wang, Xiaoxia Wang, Laurence Don Wai Luu, Shaojin Chen, Fu Jin, Shufang Wang, Xiaolan Huang, Licheng Wang, Xiaocui Zhou, Xi Chen, Xiaodai Cui, Jieqiong Li, Jun Tai, Xiong Zhu

**Affiliations:** ^1^ Experimental Research Center, Capital Institute of Pediatrics, Beijing, China; ^2^ Central & Clinical Laboratory of Sanya People’s Hospital, Sanya, China; ^3^ School of Biotechnology and Biomolecular Science, University of New South Wales, Sydney, NSW, Australia; ^4^ Nursing department of Sanya People’s Hospital, Sanya, China; ^5^ Department of Respiratory Disease, Beijing Pediatric Research Institute, Beijing Children’s Hospital, National Center for Children’s Health, Capital Medical University, Beijing, China; ^6^ Department of Otolaryngology, Head and Neck Surgery, Children’s Hospital Capital Institute of Pediatrics, Beijing, China

**Keywords:** COVID-19, SARS-CoV-2, CoronaVac, proteomics, metabolomics, immune response.

## Abstract

CoronaVac (Sinovac), an inactivated vaccine for SARS-CoV-2, has been widely used for immunization. However, analysis of the underlying molecular mechanisms driving CoronaVac-induced immunity is still limited. Here, we applied a systems biology approach to understand the mechanisms behind the adaptive immune response to CoronaVac in a cohort of 50 volunteers immunized with 2 doses of CoronaVac. Vaccination with CoronaVac led to an integrated immune response that included several effector arms of the adaptive immune system including specific IgM/IgG, humoral response and other immune response, as well as the innate immune system as shown by complement activation. Metabolites associated with immunity were also identified implicating the role of metabolites in the humoral response, complement activation and other immune response. Networks associated with the TCA cycle and amino acids metabolic pathways, such as phenylalanine metabolism, phenylalanine, tyrosine and tryptophan biosynthesis, and glycine, serine and threonine metabolism were tightly coupled with immunity. Critically, we constructed a multifactorial response network (MRN) to analyze the underlying interactions and compared the signatures affected by CoronaVac immunization and SARS-CoV-2 infection to further identify immune signatures and related metabolic pathways altered by CoronaVac immunization. These results help us to understand the host response to vaccination of CoronaVac and highlight the utility of a systems biology approach in defining molecular correlates of protection to vaccination.

## Introduction

The ongoing coronavirus disease 19 (COVID-19) pandemic, caused by severe acute respiratory syndrome coronavirus 2 (SARS-CoV-2), is an unprecedented global threat leading to high morbidity and mortality worldwide (1). Since the outbreak began, researchers from around the world have been trying to develop vaccines for COVID-19, with more than 44 candidate vaccines in the clinical development stage and another 151 vaccines in preclinical evaluation as of February, 2021 (2). CoronaVac (Sinovac Life Sciences, Beijing, China), an inactivated vaccine against COVID-19 has shown good immunogenicity in mice, rats, and non-human primates (3, 4). After preclinical evaluation, CoronaVac, approved by the WHO recently, has been widely used in China and other countries to immunize different populations, including children and adolescents aged 3-17 years old, adults aged 18-59, and adults aged 60 years and older (5).

Although the immunogenicity of CoronaVac has been assessed in large clinical trials involving thousands of subjects, the underlying molecular processes and cellular mechanisms by which biological messages stimulate the immune response remains poorly understood (3, 4). Previous analysis of COVID-19 vaccines has mainly focused on evaluatingimmunogenicity, as well as characterizing immune cell types and/or cytokines (3, 4, 6). Protective immunity induced by vaccines not only involves the response of the innate and adaptive immune cells, but also induces profound changes in cellular proteomic and metabolic pathways, increasing the capacity of these immune cells to respond to secondary stimulation. Systems vaccinology, which uses high-throughput cellular and molecular omics technologies, allows the immune response to be comprehensively studied to increase our understanding of vaccine-induced immunity (7–9). Being able to quickly determine vaccine immunogenicity, and specific protein and metabolite changes would aid in controlling epidemics and pandemics when speed is a critical factor.

Blood proteomics and metabolomics have provided valuable insights into the early events of vaccine-induced immune response (10–13). For example, proteomic signatures after vaccination have been used to predict vaccine-induced T cell responses in multiple studies, and different classes of vaccines have been shown to induce distinct protein expression patterns (12). The coordinated action of the immune system induced by vaccines resembles a social network. This enables complex immunological tasks to be performed beyond the sum of the functions of individual immune cells (9, 10). Furthermore, increasing evidence have linked trained immunity to epigenetic and metabolic regulation that involve a number of central cellular metabolic pathways such as glycolysis, oxidative phosphorylation, as well as fatty acids and cholesterol-synthesis pathways (14–16). Metabolic rewiring is a crucial step for the induction of trained immunity after immunization, but many questions remain including which metabolic pathways are involved (e.g. the role of pentose phosphate pathway or reactive oxygen species metabolism), what immune cells are affected and what specific effects do these metabolic changes have in the affected immune cells (17). Taken together, proteomics and metabolic studies contribute to the emerging field of systems vaccinology and open up new ways to understand the molecular mechanisms of vaccine-induced immunity.

Beside immunogenicity, inflammation evaluation is another important parameter for vaccine evaluation. Recently, a growing body of clinical data suggests that proteomic and metabolic dysregulation are associated with COVID-19 pathogenesis (18, 19). For example, acute phase proteins (APPs) including serum amyloid A-1 (SAA1), SAA2, SAA4 and C-reactive protein (CRP) were increased in severe COVID-19 patients, indicating activation of inflammation and the complement system (18). This leads to enhanced cytokine and chemokine production, potentially contributing to ‘cytokine storm’, and increases recruitment of macrophages from peripheral blood, which may result in acute lung injury (20). In contrast to infection, the inflammatory response induced by the inactivated vaccine, CoronaVac, should be kept at an appropriate level while still promoting immune cell activation. For this reason, proteomic and metabolomic analysis of vaccine immunized subjects are essential in evaluating the inflammation of CoronaVac.

To enhance our understanding of the mechanisms behind CoronaVac-induced protection to SARS-CoV-2, we combined multi-omics data, including plasma proteomics, metabolomics, cytokine analysis, and specific IgM/IgG, coupled with computational approaches to construct a global overview of the immune response induced by CoronaVac. The goal of this study is 1) to evaluate the plasma proteomic and metabolomic phenotypes of adaptive immunity to CoronaVac, 2) to delineate the molecular mechanisms that generate protective immunity, and 3) to evaluate if there were “cytokine storm” and excessive inflammation induced by CoronaVac. Understanding how proteins and metabolites affect vaccine immune response has important implications for increasing vaccine immunogenicity and offering new insights into the molecular mechanisms of protection from SARS-CoV-2 vaccines.

## Material and Methods

### Experimental Design and Participant Recruitment

**Figure 1:**
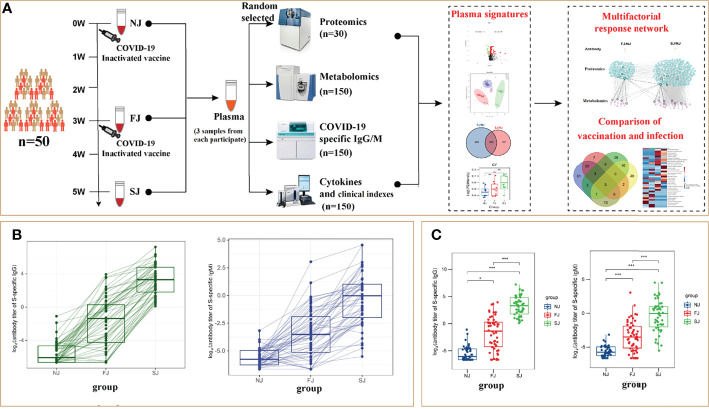
CoronaVac Study Overview and Antibody Expression. **(A)** Study overview. Fifty subjects were recruited. Different samples of each subject were taken at NJ (baseline, day 0), FJ (about 21 days after first immunization) and SJ (about 14 days after second immunization). Plasma proteomics, metabolomics, SARS-CoV-2-S-specific antibody titers, cytokines and clinical parameters were analyzed to construct an integrated network. **(B)** Representative SARS-CoV-2 S-specific IgG and IgM in each subject. **(C)** Average levels of SARS-CoV-2 S-specific IgG and IgM in each group. The Y-axis labels of Figure **(B**, **C)** were Log_2_ (antibody titer of S-specific IgG or IgM). Statistical significance was determined by two-sided paired Welch’s t test. *p < 0.05; ***p < 0.001.

Between January and April 2021, fifty participants, aged 21 to 56, were enrolled in Sanya People’s Hospital and immunized with CoronaVac. Written informed consent was obtained from each subject and protocols were approved by Institutional Review Boards of Sayan People’s Hospital. The detailed descriptions including the sampling date for each participant are shown in **Table S1**. Participants received two doses of CoronaVac and were vaccinated at 21-33 days (d) intervals. The subjects’ blood samples were collected at baseline (non-injection time point, NJ) prior to vaccination and at about 21 d after the first injection (FJ) and about 14 d after the second injection (SJ) time point, respectively. Throughout the course of this study, we measured SARS-CoV-2-S-specific antibody titers, cytokines and clinical parameters. Plasma proteomics and metabolomics were also analyzed to obtain systems vaccinology data. As a result, each subject was profiled by multiple technologies in a time series (**Figure 1A**). This rich collection of immune profiles, including high-dimensional data from proteomics and metabolomics, provided a unique opportunity to construct an integrated network of the immune response to CoronaVac in humans. For this study, we firstly present each data type separately. Second, the integrative analysis is presented in a framework of a ‘‘multifactorial response network’’ (MRN). Then, we compared the proteomic and metabolomic signatures affected by CoronaVac vaccination and SARS-CoV-2 infection. Besides, the “cytokine storm” and “the change of clinical laboratory-related factors” were also evaluated. The underling mechanisms and pathways related to CoronaVac-induced immune response were interpreted through a comprehensive analysis.

### COVID-19-Specific IgM/IgG ELISA

The S-specific IgG and IgM were detected using a chemiluminescence quantitative kit (Auto Biotechnology, Zhengzhou, China). Plates were coated with either SARS-CoV-2 recombinant antigens or mouse anti-human IgM monoclonal antibody. Ten μL of sample, 20 μL of microparticle solution and 100 μL of sample diluent were mixed and incubated for 37 min at 37°C. The plates were then washed and enzyme conjugates were added, and incubated for 17 min at 37°C. Plates were washed and chemiluminescence developed using 50 μL Chemiluminescent Substrate A and 50 μL of Chemiluminescent Substrate B. The antibody titer was measured using the AutoLumo A2000 Plus. Results with S/CO≥1.00 were considered positive while S/CO<1.00 were considered negative.

### Evaluation of Clinical Characteristics and Markers

Complete information, including count and proportion of blood cells [white blood cells (WBC), red blood cell (RBC), neutrophils (Neu), lymphocyte (Lym), eosinophils (Eos), monocytes (Mon), basophils (Bas)], hemoglobin-related clinical indicators including hemoglobin (HGB), hematocrit (HCT), mean corpuscular volume (MCV), mean corpuscular hemoglobin (MCH), mean corpuscular hemoglobin concentration (MCHC), red blood cell distribution width-coefficient of variation (RDW-CV), red blood cell distribution width-standard deviation (RDW-SD), and platelet-related clinical indicators including platelet (PLT), platelet volume distribution width (PDW), plateletcrit (PCT) were analyzed using the Sysmex XE-2100 (Sysmex Corporation).

### Multiplex Cytokine Assays

Concentrations of interferon (IFN)-γ, tumor necrosis factor (TNF)-α, granulocyte-macrophage colony stimulating factor (GM-CSF), Interleukin (IL)-1β, IL-2, IL-4, IL-5, IL-6 IL-12, IL-13 and IL-18 in plasma were determined using a bead-based, 11-plex Th1/Th2 human ProcartaPlex immunoassay (Thermo Fisher Scientific) according to the manufacturer’s instructions. Fluorescence was measured with a Luminex 200 system (Luminex Corporation) and analyzed with ProcartaPlex Analyst 1.0 software (Thermo Fisher Scientific). Only cytokines above the limit of detection were included for further analysis.

### Plasma Proteomics

Ten of the fifty subjects were randomly selected for plasma proteomic analysis (**Table S1**). Three samples were collected from each subjects. To remove highly abundant interfering proteins in human plasma, a multiple-affinity removal system liquid chromatography (LC) column (High Select™ Top14 Abundant Protein Depletion Mini Spin Columns; Thermo Fisher Technologies, Santa Clara, CA, USA) was used. Briefly, plasma samples loaded onto a multiple-affinity removal system LC column were eluted into fractions which contained low-abundance proteins while highly abundant proteins were removed. The concentration of plasma proteins was measured and 50 µg protein samples were prepared for mass spectrometry (MS) analysis. Biological replicates were performed in this study.

Plasma from each sample was lysed in 100 μL lysis buffer (8 M urea in 100 mM triethylammonium bicarbonate, TEAB) at 25°C for 30 min. The lysates were reduced using 5 mM Tris (2-carboxyethyl) phosphine (Pierce, Rockford, IL, USA) and incubated at 37°C for 30 min with shaking (300 rpm). Then, 15 mM Iodoacetamide (Sigma-Aldrich, St. Louis, MO, USA) was added for alkylation. Proteins were trypsin digested overnight at 37°C. Mass spectrometry-grade trypsin gold (Promega, Madison, WI, USA) was used with an enzyme-to-protein ratio of 1:50. The dried peptides were dissolved in 20 μL loading buffer (1% formic acid, FA; 1% acetonitrile, ACN). Ten μL of sample was used for LC-MS/MS analysis on an Orbitrap Fusion Lumos in data dependent acquisition (DDA) mode coupled with Ultimate 3000 (Thermo Fisher Scientific, Waltham, MA, USA). The samples were loaded and separated by a C18 trap column (3mm 0.10×20mm), packed with C18 reverse phase particle (1.9mm 0.15×120mm, Phenomenex, Torrance, California, USA).

The parameters for MS detection were as follows: full MS survey scans were performed in the ultra-high-field Orbitrap analyzer at a resolution of 120,000 and trap size of 500,000 ions over a mass range from 300 to 1400 m/z. MS/MS scan were detected in IonTrap and the 20 most intense peptide ions with charge states 2 to 7 were subjected to fragmentation *via* higher energy collision-induced dissociation (5×10^3^ AGC target, 35 ms maximum ion time). The resultant mass spectrometry data were analyzed using Maxquant (Version 1.6.17) and the protein search database used was the *Homo sapiens* FASTA database downloaded from UniprotKB (UP000005640.fasta). The following search parameters were used for Maxquant: precursor ion mass tolerance was set at 20 ppm; full cleavage by trypsin was selected; a maximum of two missed cleavages was allowed; static modifications were set to carbamidomethylation of cysteine, and variable modifications were set to oxidation of methionine and acetylation of peptides’ N-termini. The remaining parameters followed the default Maxquant setup. For protein identification, the following criteria was used: (1) peptide length ≥6 amino acids; (2) FDR ≤1% at the PSM, peptide and protein levels. Peptides were quantified using the peak area derived from their MS1 intensity. The intensity of unique and razor peptides was used to calculate the protein intensity.

### Plasma Metabolomics

A total of 150 plasma samples, collected from fifty participants, were selected for metabolomic analysis. To ensure data quality for metabolic profiling, pooled quality control (QC) samples were prepared by mixing equal amounts of plasma (0.75 mL) from 150 samples. The pretreatment of the QC samples was performed in parallel and was the same as the other samples. The QC samples were evenly inserted between each set of runs to monitor the stability of the large-scale analysis.

Plasma samples were extracted by adding 400 μL of MeOH/ACN (1:1, v/v) solvent mixture to 100 μL of plasma (2:2:1 ratio). The mixtures were shaken vigorously for 5 min and incubated for 1 h at -20°C. Samples were then centrifuged for 10 minutes at 13,500g at 4°C and the supernatant was transferred to a new centrifuge tube. Each supernatant was divided into three fractions: two for reverse-phase/ultra-performance liquid chromatography (RP/UPLC)-MS/MS methods with positive ion-mode electrospray ionization (ESI) and negative-ion mode ESI, and one for hydrophilic interaction liquid chromatography (HILIC)/UPLC-MS/MS with positive-ion mode ESI.

All UPLC-MS/MS methods used the ACQUITY 2D UPLC system (Waters, Milford, MA, USA) and Q-Exactive Quadrupole-Orbitrap (QE, Thermo Fisher Scientific™, San Jose, USA) and TripleTOF 5600+ (AB SCIEX, MA, USA) with ESI source and mass analyzer. In the UPLC-MS/MS method, the QE was operated under ESI coupled with a C18 column (UPLC BEH C18, 2.1×100 mm, 1.7 μm; Waters). The mobile solutions used in the gradient elution were water and methanol containing 0.1% FA. When the QE was operated under negative ESI mode, the UPLC method used a C18 column eluted with mobile solutions containing methanol and water in 6.5 mM ammonium bicarbonate at pH 8. The UPLC column used in the hydrophilic interaction method was a HILIC column (UPLC BEH Amide, 2.1×150 mm, 1.7 μm; Waters), and the mobile solutions consisted of water and acetonitrile with 9 mM ammonium formate at pH 8.0; the TripleTOF 5600+ was operated under positive ESI mode. The mass spectrometry analysis alternated between MS and data-dependent MS2 scans using dynamic exclusion. The scan range was 70-1,000 m/z. After raw data pre-processing, peak finding/alignment, and peak annotation using MSDIAL software, metabolite identifications were supported by matching the retention time, accurate mass, and MS/MS fragmentation data to MSDIAL software database and online MS/MS libraries (Human Metabolome Database).

### Statistical Analysis

Missing values of proteomic data were substituted by perseus 1.6.15.0; R3.6. Missing values of metabolomic data were substituted with 1/5 the minimal value. For each comparison, the log_2_ fold-change (log_2_ FC) was calculated by averaging the paired fold change for each participant. A two-sided paired Welch’s t test was also performed. Statistical significance and differentially expressed proteins or metabolites were assigned as |log_2_(FC)| >0.25 and p value <0.05.

Partial least squares-discriminate analysis (PLS-DA) for proteomics and orthogonal partial least squares discrimination analysis (OPLS-DA) for metabolomics and were conducted using MetaboAnalyst 5.0. Volcano plots were calculated using a combination of FC and paired Welch’s t test. The intensity data of these regions were used in box-plot analysis. Heatmaps were displayed using the Multi Experiment Viewer software (MeV, version 4.7.4). Radar maps were made using R packets. A Web-accessible resource was used to determine the over-representation of gene ontology (GO) categories. Signaling pathway analysis was performed by the Kyoto Encyclopedia of Genes and Genome (KEGG) database. Pathway analysis and visualization were performed using the Metaboanalyst 5.0 web portal. Connected networks of the proteins were built and analyzed in BINGO. Relationship of proteins and metabolites was constructed by MetaboAnalyst. Humoral immune related proteins are thought to be associated with IgG antibodies. The correlation between IgG and metabolites was analyzed using Pearson’s correlation (p<0.05). Ultimately, these interrelated DEPs, DEMs and their relationships were selected to construct a MRN by Cytoscape v3.8.2.

## Results

### CoronaVac-Induced Antibody Responses

To ensure the effectiveness of vaccination, we assessed the antibody response to SARS-CoV-2 induced by CoronaVac. At baseline, none of the participants had any detectable S-specific IgG and IgM antibodies. The seroconversion rates of IgG were 18 (36%) at FJ point versus 50 (100%) of 50 participants at SJ point, and the seroconversion rates of IgM were 2 (4%) at FJ point versus 25 (50%) of 50 participants at SJ point. The dynamic changes of IgG and IgM to SARS-CoV-2 are shown in **Figure 1B** and illustrates that the IgG antibody levels did not significantly increase until after the second dose of the vaccine. Additionally, as shown in **Figure 1C**, the levels of IgM and IgG were 2.562 ± 4.806 s/co and 19.691 ± 26.86 s/co at SJ point, significantly higher than that at FJ point (IgM 0.388 ± 1.202 s/co, IgG 1.667 ± 3.21 s/co) and the baseline (IgM 0.024 ± 0.017 s/co, IgG 0.039 ± 0.075 s/co). Taken together, these results show that CoronaVac induced antibody responses in all subjects involved in this study. This was consistent with the increased antibody levels reported in clinical trials (3, 4).

### Plasma Proteomic Signatures After CoronaVac Vaccination

Based on the LC-MS/MS data, a total of 5064 peptides (**Figure S1A**) and 387 proteins (**Figure S1B**) were identified. We applied the PLS-DA analysis (**Figure S2A**) and volcano plots (**Figures S2B, C**) to show the different proteins. Accordingly, 56 and 80 different expressed proteins (DEPs) were observed in FJ and SJ group compared to baseline (NJ), respectively. Among them, 20 DEPs were overlapping and a total of 116 DEPs were identified in vaccinated samples (**Figure S2D**, and **Table S2**).

**Figure 2:**
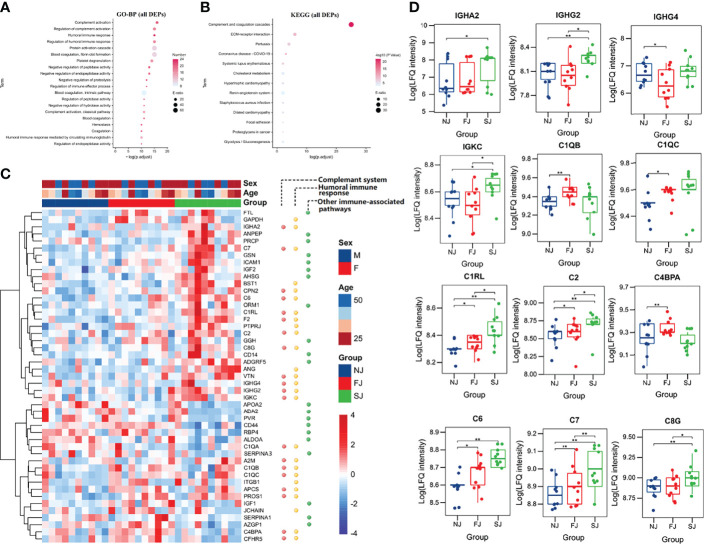
Enrichment and Distribution of Immune Related Proteins. **(A)** GO-BP analysis of the total DEPs from FJ vs NJ and SJ vs NJ. Top 20 GO-BP terms were expressed. **(B)** KEGG pathway analysis of the total DEPs from FJ vs NJ and SJ vs NJ. Top 12 KEGG terms were expressed. **(C)** Heatmap of selected proteins from three enriched pathways: complement response, humoral immune response, and other immune associated-pathways. **(D)** The expression level comparison of humoral immune and complement related proteins with significant differences. Statistical significance was determined by two-sided paired Welch’s t test. *p < 0.05; **p < 0.01.

As shown in **Figure 2A**, biological processes terms of GO (GO-BP) for the 116 DEPs were highly enriched in processes involved in known immune-related functions, such as complement activation, regulation of complement activation, humoral immune response, and regulation of humoral immune response. KEGG analysis of these DEPs indicted that complement and coagulation cascades was the top most significant pathway impacted and associated with vaccination (**Figure 2B**). Moreover, GO-BP (**Figures S2E, F**) and KEGG analysis (**Figures S2G, H**) of DEPs from FJ vs NJ and SJ vs NJ also showed similar enrichment. This suggests that the humoral and complement response participate in the immunity induced by CoronaVac.

### Evidence of Humoral and Complement Response Activation After CoronaVac Immunization

An important goal of systems vaccinology is to evaluate the adaptive response and innate immunity to vaccination. Here, proteomics data showed that adaptive immunity, especially humoral and complement response were activated by vaccination. As shown in **Figure 2C**, 47 DEPs belonged to three major pathways: activation of the complement response, humoral immune response, and other immune-associated pathways. At the SJ time point, multiple immunoglobulin heavy chains, including IGHA2, IGHG2, IGHG4, and IGKC were highly upregulated (**Figure 2D**). Increased expression of IgG in the plasma of vaccinated subjects also supports this finding (**Figure 1B**).

Complements were reported to be a central regulator for adaptive immune responses (21). Although several recent studies have implicated complement activity or impairment in severe COVID-19 patients, the potential involvement of complement factors in protective immunity has been largely ignored (22). In this study, we found that several complements, including C1QB, C1QC, C1RL, C2, C4BPA, C6, C7, and C8G were significantly increased at FJ and/or SJ time point (**Figure 2D**).

In addition, other immune associated proteins were also observed to be increased in vaccine immunized subjects. For instance, the level of ICAM-1, essential for mediating T cell migration and activation (23, 24), was significantly increased in immunized samples. Human monocyte differentiation antigen CD14, a pattern recognition receptor for enhancing the innate immune response (25), was significantly increased after immunization (**Figure 2C**). Collectively, our results demonstrate the activation of adaptive responses and complement responses was observed after CoronaVac vaccination.

### Correlation Analysis of the Immune-Related Proteins

**Figure 3:**
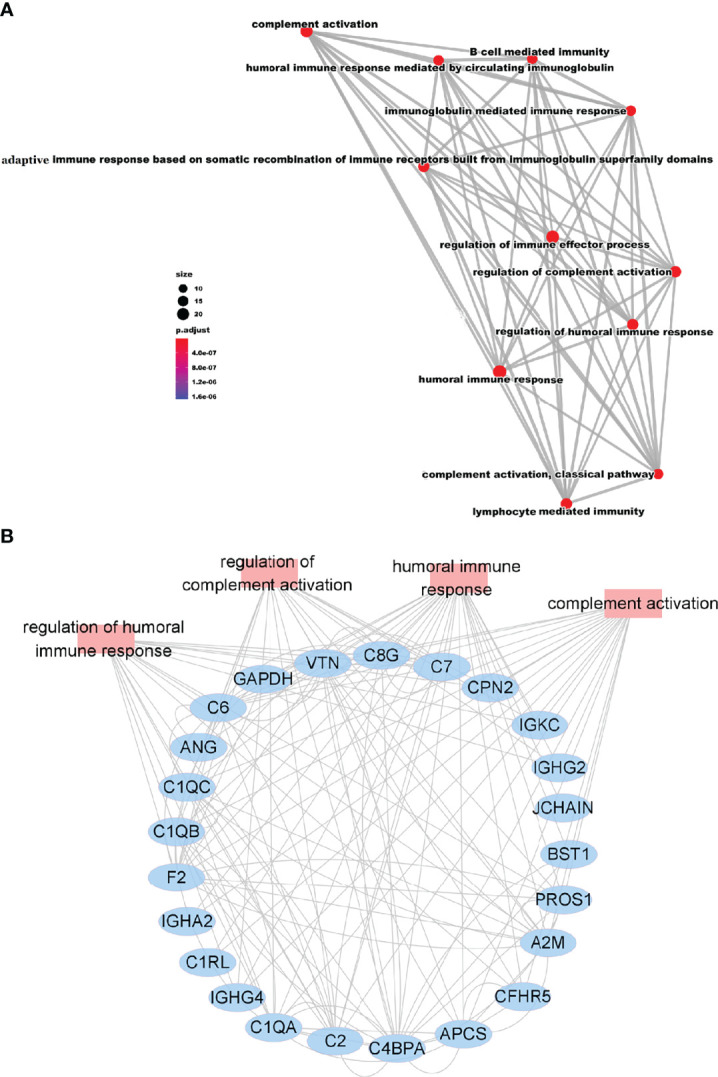
Correlation Analysis of the Immune-Related Proteins. **(A)** Network of GO modules related to the immune response defined by BINGO. **(B)** Interaction diagram of proteins involved in the humoral immune response, regulation of complement activation, humoral immune response, and regulation of complement activation.

To further understand the functions and interactions of DEPs induced by CoronaVac vaccination, we categorized these proteins based on GO-BP using the BINGO of Cytoscape (**Figure 3A**). All of the modules clustered into one group, reflecting their functional lineage relationship.

Among the 11 resulting functional modules, humoral regulated proteins were enriched for “regulation of humoral immune response”, “B cell mediated immunity”, “humoral immune response” and “immunoglobulin mediated immune response”, and created a complex network (**Figure 3A**). Humoral response was also tightly connected with complement-related modules such as “complement activation”, “regulation of complement activation”, and “complement activation-classical pathway” (**Figure 3A**). Furthermore, the network of lymphocyte-related immunity, containing immunoglobulin proteins, was also significantly connected with the humoral and complement response network (**Figure 3A**). The specialized function of complement, comprised of many proteins, was highly enriched for “complement activation” and “regulation of complement activation”. Notably, the proteins belonging to these modules were also connected with each other (**Figure 3B**). Together, these results demonstrate that DEPs involved in adaptive and innate immunity formed a complex interactive network which allowed us to further analyze the host response to CoronaVac, and how these responses shape the development of adaptive immunity.

### Plasma Metabolomic Signatures Induced by CoronaVac

We identified 1190 metabolites. To ensure reliable results, the OPLS-DA score plot (**Figure S3A**) and the relative standard deviation (RSD) in the QC samples (**Figure S3B**) were applied. Volcano plots from the metabolomic analysis highlighted the different expressed metabolites (DEMs) identified in FJ (**Figure S4A**) and SJ group (**Figure S4B**) compared to NJ group, respectively. In this study, we observed 742 and 638 DEMs in FJ and SJ group compared to the baseline (NJ), respectively. Collectively, 499 DEMs were overlapping and a total of 881 DEMs were identified in vaccinated samples (**Figure S4C** and **Table S3**).

Next, KEGG analysis were applied for pathway analysis of DEMs from FJ vs NJ (**Figure S4D**) and SJ vs NJ (**Figure S4E**), respectively. Besides, pathway analysis of all 881 DEMs were also analyzed to indicate the pathway associated with the immunization. As shown in **Figure S4F**, KEGG of the total DEMs revealed CoronaVac immunization had a significant impact on aminoacyl-tRNA biosynthesis, TCA cycle and amino acid metabolism, such as valine, leucine and isoleucine biosynthesis, phenylalanine metabolism, tryptophan metabolism, and glycine, serine and threonine metabolism. Collectively, these results revealed distinct metabolomic signatures in the immune response to CoronaVac which provided key insights into vaccine-induced antiviral responses.

### Metabolites Associated With the Humoral and Complement Immune Response Are Altered After CoronaVac Immunization

**Figure 4:**
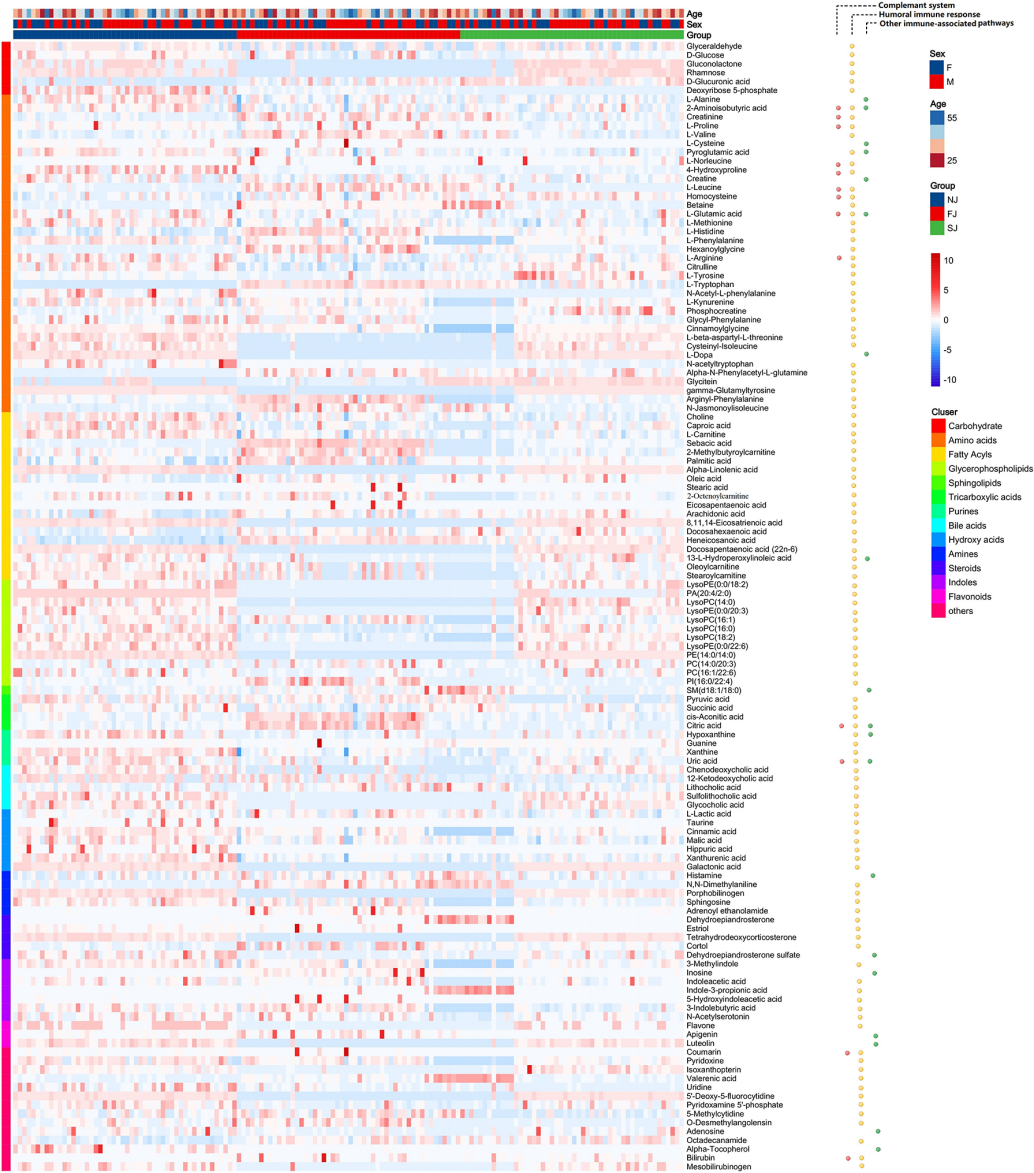
Enrichment and Distribution of Immune Related Metabolites. Heatmap of the DEMs associated with three enriched pathways: complement system, humoral immune response, and other immune associated-pathways.

To elucidate the role of metabolites in vaccine immunity, we analyzed the correlation of DEPs and immune related proteins (**Figures S5A-C**), as well as the correlation of DEPs expressions and IgG levels (**Figure S5D**). Consistent with our proteomic analysis, 128 significant DEMs including carbohydrates, amino acids, and several types of lipids were involved in the three enriched biological processes identified in the proteomic analysis (**Figure 4**).

**Figure 5:**
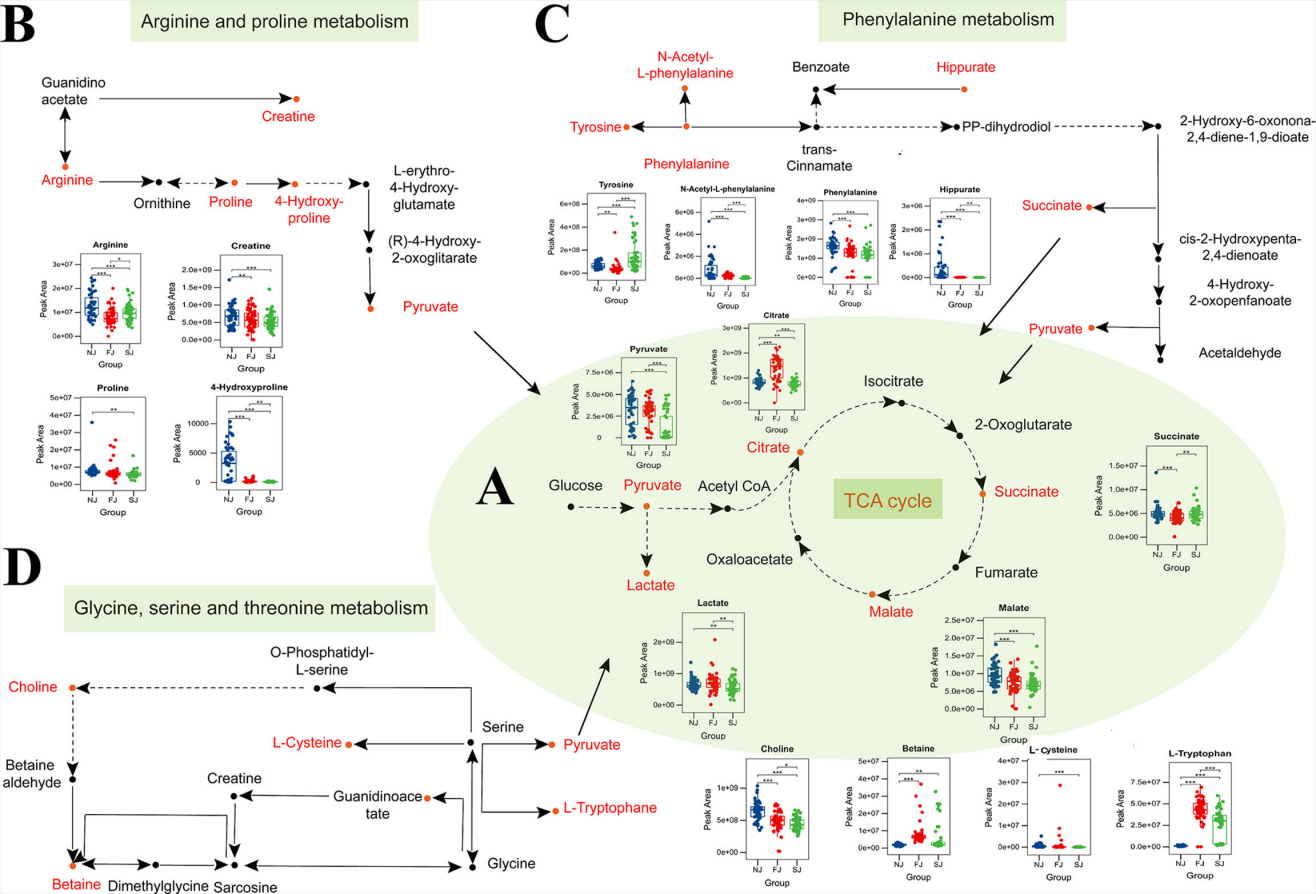
Changes in Amino Acid Levels, Carbohydrate Levels, and Their Metabolic Pathways After Vaccination. **(A)** Circulating levels of TCA metabolites in plasma. Plasma levels of metabolites involved in the TCA cycle were significantly changed when comparing vaccination samples with baseline samples. **(B)** Significant changes were seen in the levels of some intermediates of the arginine and proline metabolism pathway in plasma of vaccine-immunized samples. **(C)** Significant changes were observed in the levels of some intermediates of the phenylalanine metabolism pathway in plasma of vaccine-immunized samples. **(D)** Significant changes were observed in the levels of metabolites involved in glycine, serine and threonine metabolism pathway after vaccination. Changed metabolites after vaccination were labeled as red. Statistical significance was determined by two-sided paired Welch’s t test. *p < 0.05; **p < 0.01; ***p < 0.001.

Production of antibody after vaccination requires large quantities of amino acids and glycosylation sugars to properly build and fold antibodies (26). In this study, we found significant changes in TCA intermediary metabolites (pyruvate, citrate, succinate, malate, and lactate) in vaccine immunized samples, implying involvement of the TCA cycle in vaccination (**Figure 5A**). The balance between energy and amino acid metabolism is essential for antibody production (27) and in this study, we also identified more than 20 amino acids that were significantly altered in vaccine immunized samples compared to baseline samples (NJ). We found metabolites involved in arginine and proline metabolism were significantly changed after vaccination (**Figure 5B**). Additionally, several amino acids involved in phenylalanine metabolism were also significantly altered in vaccinated samples (**Figure 5C**). Phenylalanine metabolism is associated with humoral autoimmune diseases, which suggests an underlying function in the humoral response (28). Amino acids and metabolites involved in tryptophan metabolism were also significantly altered after CoronaVac immunization (**Figures S6A, B**). In addition, several fatty acids, essential for the plasma cell differentiation were also significantly changed in vaccinated samples (**Figure S6C**).

Glycine, serine and threonine metabolism have been reported to affect complement-mediated killing (29). In this study, L-cysteine, betaine, choline, and L-tryptophan, which are enriched in the glycine, serine and threonine metabolism pathway, were significantly changed in vaccine immunized samples (**Figure 5D**). Taken together, our data suggests that altered metabolites may be a crucial step for adaptive immunity, and further integrated analysis will increase understanding of the underlying mechanism for how CoronaVac induces protection.

### MRN Reveals a Connection Between Antibody Response, Proteins, and Metabolites Following CoronaVac Immunization

**Figure 6:**
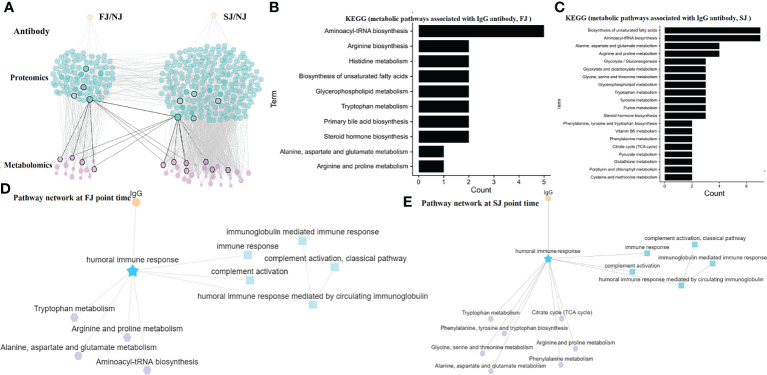
MRN analysis of Metabolomics, Proteomics, and Antibodies. **(A)** MRN consists of correlation networks using data from antibody, DEPs, and DEMs. Each node is a child network of one data type. The links between nodes were established by weight. **(B)** The top 10 pathways for metabolite networks correlated with IgG levels at FJ time point. **(C)** The top 20 pathways for metabolite networks correlated with IgG levels at SJ time point. **(D)** Connections between IgG, humoral response associated network and metabolite networks at FJ time point are shown. **(E)** Connections between IgG, humoral response associated network and metabolite networks at SJ time point are shown.

To integrate antibody, proteins and metabolites, we constructed a MRN for mechanistic analysis. The MRN had a dense network of 329 nodes and 1395 connections. This hierarchical structure allows an overview of the super-network and reveals the complex relationship of the adaptive immune response induced by CoronaVac (**Figure 6A**).

A humoral response-related network was obtained to further investigate the underlying mechanisms following vaccination. As described above, levels of IGHA2, IGHG2, IGHG4, and IGKC, correlated with IgG antibody titers, were significantly increased after vaccination (**Figure 2D**). Relationship of DEPs and DEMs was constructed by MetaboAnalyst. Besides, the expression of some DEMs were also correlated with the IgG level (**Figure S5D**). According, we selected the antibody-related metabolites to analyze the metabolic pathways associated with IgG at the FJ time point (**Figure 6B**) and at the SJ time point (**Figure 6C**) by KEGG, respectively. In details, we observed that aminoacyl-tRNA biosynthesis and amino acid metabolic pathways such as tryptophan metabolism, alanine, aspartate and glutamate metabolism, and arginine and proline metabolism, which were all significantly altered following CoronaVac immunization at FJ time point are involved in and affects the IgG antibody response (**Figure 6D**). In addition, after the second immunization, the TCA cycle, the phenylalanine metabolism, and the phenylalanine, tyrosine and tryptophan biosynthesis pathways were associated with the IgG antibody response (**Figure 6E**). Finally, the humoral immune response pathway was also connected with complement activation and complement activation-classical pathway (which also showed tight correlation with the metabolism pathways mentioned above). Taken together, these data suggest that high activity in these metabolic pathways discussed above was detrimental to the humoral immune response induced by CoronaVac and they combined a complex network in which many proteins and metabolites are involved.

### Comparison of the Proteomic Signatures Induced by CoronaVac Immunization and SARS-CoV-2 Infection

**Figure 7:**
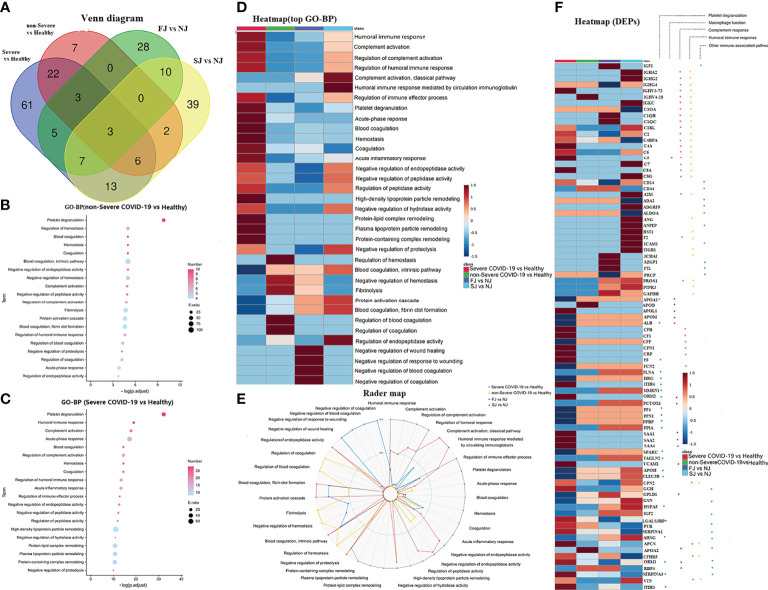
Comparison of DEPs and their enriched pathways in severe COVID-19 infection, non-Severe COVID-19 infection, FJ and SJ group. **(A)** Venn diagram showing the number of DEPs from severe COVID-19 vs healthy, non-Severe COVID-19 vs healthy, FJ vs NJ and SJ vs NJ. **(B)** GO-BP analysis of DEPs from non-Severe COVID-19 vs healthy. Top 20 GO-BP terms were expressed. **(C)** GO-BP analysis of DEPs from severe COVID-19 vs healthy. Top 20 GO-BP terms were expressed. **(D)** Heatmap of the top GO-BP terms enriched from DEPs in severe COVID-19 vs healthy, non-Severe COVID-19 vs healthy, FJ vs NJ and SJ vs NJ. **(E)** Radar map of the top GO-BP terms enriched from DEPs in severe COVID-19 vs healthy, non-Severe COVID-19 vs healthy, FJ vs NJ, and SJ vs NJ, respectively. -Log_10_ p values of GO-BP terms were used to make the radar map. **(F)** Heatmap of DEPs from vaccination and infection that were related to platelet degranulation macrophage function, complement response, humoral immune response and other immune-associated pathway.

To gain an insight into the mechanisms underlying the responses to vaccines against SARS-CoV-2, we combined data from a published paper (18) to compare differences in the proteomic signatures affected by vaccination and SARS-CoV-2 infection. Notably, DEPs in vaccination and infection were different (**Figure 7A**). Interestingly, GO-BP of DEPs after vaccination were highly enriched in processes involved in known immune-related functions such as complement activation, regulation of complement activation, humoral immune response, and regulation of humoral immune response (**Figure 2A**). In comparison, DEPs from non-severe or severe COVID-19 patients were mainly enriched in platelet degranulation, regulation of hemostasis, blood coagulation (**Figure 7B**), humoral immune response, complement activation, and acute-phase response (**Figure 7C**). Surprisingly however, several pathways related to immunity including humoral immune response, complement activation, regulation of complement activation and regulation of humoral immune response, which were induced by CoronaVac vaccination, were also strongly enriched in severe COVID-19 patients (**Figure 7D**). Enriched pathways unique to CoronaVac vaccination were complement activation-classical pathway and humoral immune response mediated by circulating immunoglobulin (**Figure 7E**).

It was reported that SARS-CoV-2 infection induced dysregulation of macrophages, platelet degranulation and complement system pathways (18). Thus, DEPs related to immune response, macrophage function, and platelet degranulation were selected for further expression analysis (**Figure 7F**). Compared to infection, many circulating immunoglobulins and complements were elevated in CoronaVac immunized samples. For example, IGHA2 and IGHG2 were significantly increased after vaccination while they were not significant changed after infection (**Figure 7F**). Both vaccines and infections lead to the activation of the complement system, but the phenotypes and underlying functions may be different. As shown in **Figure 7F**, C1QB, C1QC, and C7 were significantly increased after vaccination, while C5 and C8A were increased after infection. In addition, SARS-CoV-2 infection induced elevated acute phase proteins, including serum amyloid A (SAA)1, SAA2, SAA4, CRP, and alpha-1-antichymotrypsin (SERPINA3). As expected, except for SERPINA3 most acute phase proteins showed no changes following CoronaVac immunization. Besides, CoronaVac immunized samples did not alter the expression of platelet related proteins, such as platelet-expressing chemokines proplatelet basic protein (PPBP) and platelet factor 4 (PF4). Interestingly, proteins such as apolipoprotein H (APOH), C-type lectin domain family 3 member B (CLEC3B), and heat shock 70kDa protein 5 (HSPA5) also showed opposite trends between COVID-19 patients and CoronaVac immunized subjects (**Figure 7F**).

### Comparison of the Metabolomic Signatures Induced by CoronaVac Immunization and SARS-CoV-2 Infection

**Figure 8:**
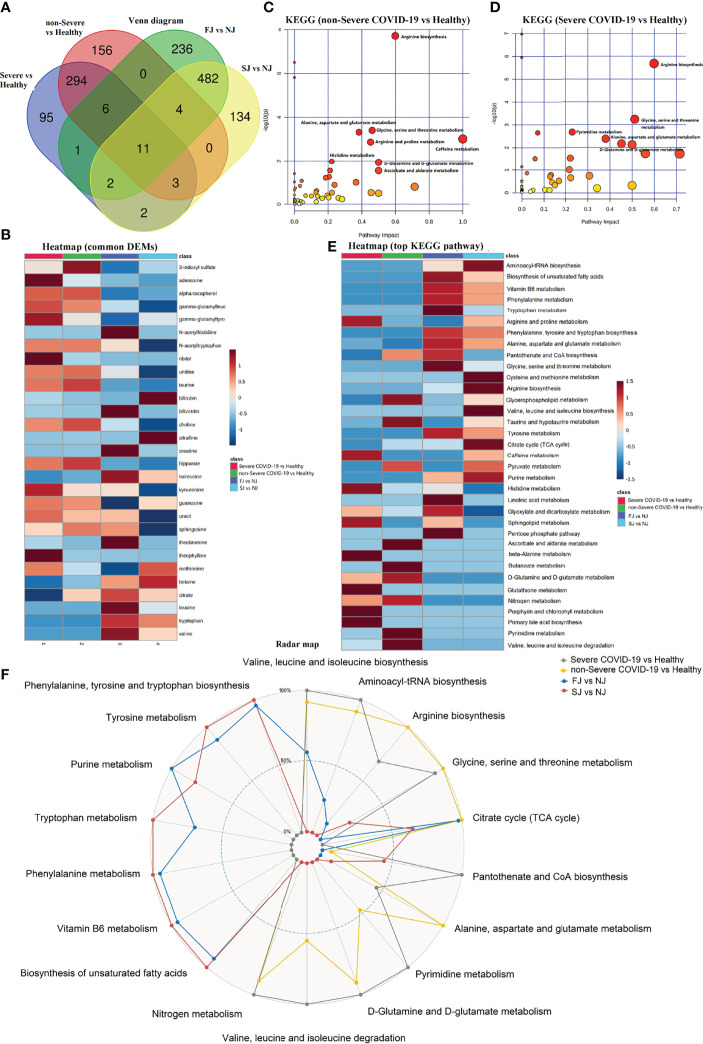
Comparison of DEMs and their enriched pathways in severe COVID-19 infection, non-Severe COVID-19 infection, FJ and SJ group. **(A)** Venn diagram showing the number of DEMs from severe COVID-19 vs healthy, non-Severe COVID-19 vs healthy, FJ vs NJ, and SJ vs NJ. **(B)** Heatmap of the common metabolites shared between CoronaVac immunization and infection. **(C)** KEGG pathway analysis of DEMs from severe COVID-19 vs healthy. **(D)** KEGG pathway analysis of DEMs from non-Severe COVID-19 vs healthy. **(E)** Heatmap of the top KEGG terms enriched from DEMs in severe COVID-19 vs healthy, non-Severe COVID-19 vs healthy, FJ vs NJ and SJ vs NJ. **(F)** Radar map of the top KEGG terms enriched from DEMs in severe COVID-19 vs healthy, non-Severe COVID-19 vs healthy, FJ vs NJ and SJ vs NJ, respectively. -Log_10_ p values of KEGG terms were used to make the radar map.

Metabolomic phenotypes induced by vaccination and infection were also significantly different (**Figure 8A**). Interestingly, there were some common DEMs identified between vaccination and infection, however, the expression trends were almost the opposite. For example, the levels of valine, tryptophan, leucine, citrate, betaine, and others were decreased in COVID-19 patients but increased in vaccination samples. Conversely, 3-indoxyl sulfate, adenosine, alpha-tocopherol, and others were increased in COVID-19 patients but decreased in vaccinationsamples (**Figure 8B**).

Similar to vaccination, the metabolomic data for COVID-19 infection also revealed a significant impact on amino acid metabolism. However, the types of amino acid metabolism pathways affected by vaccination and infection were different: COVID-19 infection mainly affected pathways involved in arginine biosynthesis, alanine, aspartate and glutamate metabolism, and glycine, serine and threonine metabolism (**Figures 8C, D**). Compared to infection, vaccination with CoronaVac significantly altered other pathways including: vitamin B6 metabolism, biosynthesis of unsaturated fatty acids, phenylalanine metabolism, and other metabolisms (**Figures 8E, F**). Altogether, vaccination caused a variety of unique proteomic and metabolomic changes compared to infection, and these differences may play an essential role in the protective mechanism induced by CoronaVac.

### Evaluation of “Cytokine Storm” and “Clinical-Related Factors”

Previous data have shown that SARS-CoV-2 infection causes a variety of disorders including “cytokine storm” and clinical laboratory-related factors (30). Thus, we evaluated these indicators in individuals after vaccination. Here, no significant differences in inflammatory cytokines were observed in immunized samples (**Table S4** and **Figure S7**); this was consistent with previous studies which also reported a lack of upregulated cytokines for subjects immunized with CoronaVac (3, 4).

Moreover, we analyzed 22 clinical measurements, including count and proportion of blood cells (WBC, RBC, Neu, Lym, Eos, Mon, Bas), hemoglobin-related clinical indicators and platelet-related clinical indicators to evaluation the clinical changes induced by CoronaVac. Compared to baseline (NJ), no significant changes except for Eos were observed in plasma from the immunized group (**Table S5** and **Figure S8**). Altogether, our data suggests that CoronaVac does not cause “cytokine storm” and excessive change of clinical-related factors in immunized subjects.

## Discussion

This study is, to our knowledge, the first study to combine multi-omics data, including plasma proteomics and metabolomics with cytokines, clinical indexes, and specific IgM/IgG for system biology analysis of CoronaVac. Previously, systems biology have been applied to identify signatures of immune responses to vaccination and have provided insights into the mechanisms of immune responses induced by different vaccines such as the live attenuated yellow fever vaccine (YF-17D) (31, 32), smallpox vaccine (33), malaria vaccine (34) and influenza vaccines (7). These insights can be used to guide novel strategies for vaccine evaluation and design.

It was observed that CoronaVac can induce quick antibody responses which may be suitable for emergency use and is of vital importance during the COVID-19 pandemic (3, 4). However, the underlying molecular mechanisms induced by CoronaVac is still a mystery. Here, we combined proteomics and metabolomics to identify the signatures associated with the immune response to CoronaVac as changes in genes, proteins and metabolites may reflect the immune status. It may also reveal the underlying molecular mechanisms involved in vaccine-induced immunity. Our results showed that IgG and other immunoglobulin components such as IGHA2, IGHG2, IGHG4, IGKC, and IGKV2-24, were significantly elevated in vaccine immunized samples. IGHA2, IGHG2 and IGHG4 are encoded by the immunoglobulin heavy chain (IGH) constant genes (*IGHC*), while IGKC and IGKV2-24 are encoded by the subgroup of immunoglobulin light chain genes (35). These results demonstrate the activation of B-cell and plasma cells after vaccination which play an essential role in antiviral immunity. In addition, the high expression of IGHG2 and IGHG4 might demonstrate that IgG2 and IgG4 are the main type of IgG induced by CoronaVac. Consistent with our report, expression of *IGHG2* and *IGKC* were also enhanced in subjects immunized with meningococcal vaccine, and correlated with immunogenicity (36). Previous multifactorial models have also revealed integrated networks on humoral immunity induced by vaccination against various other diseases (9, 12, 36, 37). For instance, herpes zoster (HZ) induced changes in genes and metabolites associated with adaptive immunity and showed that sterol metabolism was tightly coupled with immunity (9). In Li et al. (37), gene expression data was collected from five different vaccine cohorts and revealed distinct transcriptional signatures for antibody responses to different types of vaccines. The study suggested that gene expression predictors for antibody response are probably not ‘universal’ but are dependent on the type of vaccine, which is consistent with the proposal that different types of vaccines would induce similar signatures of immunogenicity. Consistent with other reports, our findings also support the humoral response as a key factor for antiviral immunity and that the immunity induced by CoronaVac showed distinctive characteristics.

Metabolites associated with humoral immunity were also revealed to play a potential role in the underlying mechanism for CoronaVac-induced immunity in this study. In our data, the essential intermediary metabolites in the TCA cycle were changed in vaccine immunized samples. These TCA cycle intermediary metabolites play a crucial role in regulating the immune system with some activating the immune response and others suppressing it (38). In cells with infection or other stresses, TCA cycle intermediates may accumulate and regulate inflammatory gene expression (39, 40). Surprisingly, we found that most of the TCA cycle intermediates including pyruvate, citrate, malate and lactate were significantly decreased after vaccination and were negatively associated with IgG. These results suggest that vaccination does not trigger a severe inflammatory response like infection does, and that balancing TCA cycle intermediates could impact the immune response. Furthermore, the balance between energy and amino acid metabolism is essential for antibody production. As the central link to energy metabolism, metabolites in the TCA cycle are also involved in different amino acid metabolic pathways. In this study, vaccination also caused changes in amino acids and amino acid metabolic pathways such as arginine and proline metabolism, phenylalanine metabolism and glycine, serine and threonine metabolism. Although most of the metabolites were reported to be associated with antibody production, whether these amino acid metabolites also have other immunological roles after vaccination requires further investigation (27–29).

Complements can also be activated by antigen-specific antibodies and therefore, can contribute to the adaptive immune responses (41). However, few studies have focused on vaccine-induced complement responses. There have been several reports on the role of complement in COVID-19 disease in humans, however all were focused on innate complement activation that occurs during acute infection (18, 42). These studies have generally concluded that excess complement activity can contribute to severe disease pathology. Importantly, the potential involvement of complement factors in protective immunity has been largely ignored for SARS-CoV-2 but has been defined for other viruses, bacteria, and protozoa. Here, our results highlight that several kinds of complements were significantly increased after vaccination, implying that it might play a protective role and are consistent with Kurtovic’s viewpoint (22). In other diseases, subjects immunized with the yellow fever vaccine, YF-17D, also showed activation of the complement system. Consistent with our results, complements including C1QB were also found to be upregulated in blood cells early after yellow fever (YF17D) vaccination (31). C3a, a product of the classical complement enzymatic pathway was increased at day 7 after immunization (32). In addition, immunity to many viral and nonviral pathogens relies on antibodies and antibody-mediated neutralization. This has been demonstrated *in vitro* for the human pathogens; West Nile virus (43), Nipah virus (44), and others using a combination of human and non-human antibodies and complements. It has also been suggested that COVID-19 patients with mild disease generally report normal serum concentrations of complement proteins, which suggests that these immune mediators may be able to contribute to immunity and reduce disease severity (45). In line with this, an examination of >6000 COVID-19 patients found that individuals with a dysregulated complement system were more prone to developing severe disease than those with a healthy complement system (46). Furthermore, distinct components of the complement pathway were found to be essential for activating the innate immune response, including IFN-stimulated responsive element and nuclear factor-κB reporters, against viral infection (47). Therefore, for most individuals, complement activation might contribute to reduced disease severity, whereas for a smaller percentage of individuals, the complement system might be dysregulated and associated with increased susceptibility to severe disease. The implications for antibody-complement interactions in virus neutralization and immunity should be further investigated and may have important implications for antibody-based vaccination strategies against SARS-CoV-2.

According to a phase 1/2 clinical trial, no vaccine-related serious adverse events were reported (3, 4). Similarly, no “cytokine storm” and changes in clinical indexes, except for slightly elevated eosinophils were observed after vaccination in this study. Moreover, most acute phase proteins, such as SAA1, SAA2, SAA4, CRP, SERPINA3, and SAP/APCS, which were increased in severe COVID-19 patients were not changed in our CoronaVac immunized samples. The plasma proteomic and metabolic signatures of vaccine immunized samples were also different from that of COVID-19 patients.

It should be noted that although secreted proteins and metabolites can directly reflect the immune status, they contain less information than the transcriptome and will be analyzed in future studies. Additionally, this was a single-center prospective study with a relatively small sample size and missing values, which are common in LC-MS data and may affect data interpretation in studies with smaller sample sizes. Due to the limitation of screening criteria and data sources, we only compared with one infection study. Therefore, future large-sized cohort studies are warranted to confirm the findings in this study.

In conclusion, our systems vaccinology study showed that CoronaVac immunization induced humoral immune responses against SARS-CoV-2. These results support the approval for emergency use of CoronaVac in China. The DEPs and DEMs identified formed a complex network that resulted in vaccine-induced antiviral immunity. The MRN analysis and the comparison between CoronaVac vaccination and SARS-CoV-2 infection indicated that humoral and complement responses as well as several metabolic pathways, including the TCA cycle, phenylalanine metabolism, tryptophan metabolism, arginine, proline metabolism and fatty acids pathways, might be essential for protective immunity induced by CoronaVac.

## Data Availability Statement

The datasets presented in this study can be found in online repositories. The names of the repository/repositories and accession number(s) can be found below:

ProteomeXchange database under accession number PXD032127 (http://proteomecentral.proteomexchange.org/cgi/GetDataset?ID=PXD032127).

## Ethics Statement

The studies involving human participants were reviewed and approved by Ethics Committee of the Sanya People’s Hospital (SYPH-2021-26). The patients/participants provided their written informed consent to participate in this study.

## Author Contributions

YW conceived and designed the study. YW, JT and XZ supervised this project. XW, SC, FJ, SW, XH, LW, XCZ, XC, XDC, YW and JL performed the experiments. YW, XW, JL, JT and XZ contributed the reagents, materials, and analysis tools. YW and JL performed the software. JL and YW drafted the original paper. LL, JL and YW revised and edited this paper. YW, JT and XZ reviewed the paper. All authors contributed to the article and approved the submitted version.

## Funding

This work was supported by grants from National Key Research and Development Program of China (Grant Nos. 2021YFC2301101, 2021YFC2301102), State Key Laboratory of Infectious Disease Prevention and Control (2020SKLID303), Public Service Development and Reform Pilot Project of Beijing Medical Research Institute (BMR2019-11), National Natural Science Foundation of China (81970900) and Beijing Social Science Foundation Project (19GLB033).

## Conflict of Interest

The authors declare that the research was conducted in the absence of any commercial or financial relationships that could be construed as a potential conflict of interest.

## Publisher’s Note

All claims expressed in this article are solely those of the authors and do not necessarily represent those of their affiliated organizations, or those of the publisher, the editors and the reviewers. Any product that may be evaluated in this article, or claim that may be made by its manufacturer, is not guaranteed or endorsed by the publisher.

## Supplementary Material

Supplementary Figure 1 Quality Control of Proteomic and Metabolomic Data. **(A)** The distribution of the numbers of quantified peptides in the 30 plasma samples. **(B)** The distribution of the numbers of quantified proteins in the 30 plasma samples.

Supplementary Figure 2 Differentially Expressed Proteins in Different Groups, Related to Figure 2. **(A)** Partial Least Squares Discriminant Analysis (PLS-DA) was used to compare the proteins of the different groups. **(B)** Volcano plot comparing protein expression in FJ vs NJ. **(C)** Volcano plot comparing protein expression in SJ vs NJ. Proteins with log2 (fold-change) above 0.25 or below -0.25 and p value <0.05 were considered to be significantly DEPs. Number of significantly down- (green) and up- (red) regulated proteins are shown on top. **(D)** Venn diagram of the number of DEPs. **(E)** GO-BP analysis of the DEPs from FJ vs NJ. **(F)** KEGG pathway analysis of the DEPs from FJ vs NJ. **(G)** GO-BP analysis of the DEPs from SJ vs NJ. **(H)** KEGG pathway analysis of the DEPs from FJ vs NJ. Top GO-BP and KEGG terms were expressed.

Supplementary Figure 3 Quality Control of Metabolomics. **(A)** Reliability investigation of the analytical method using QC samples (n = 5). Score plot of Orthogonal Partial Least Squares Discrimination Analysis (OPLS-DA). **(B)** RSD% of all detected variables. Number (%): percentage of number of variables. Sum of intensity (%): percentage of total response of variables.

Supplementary Figure 4 Differentially Expressed Metabolites in Different Groups, Related to Figure 4. **(A)** Volcano plot comparing metabolite expression in FJ vs NJ. **(B)** Volcano plot comparing metabolite expression in SJ vs NJ. Metabolites with log2 (fold-change) above 0.25 or below 0.25 with p value <0.05 were considered to be DEMs. Number of significantly down- (green) and up- (red) regulated proteins are shown on top. **(C)** Venn diagram showing the number of DEMs. **(D)** KEGG pathway analysis of the DEMs from FJ vs NJ. **(D)** KEGG pathway analysis of the DEMs from SJ vs NJ. (E) KEGG pathway analysis of the total DEMs from FJ vs NJ and SJ vs NJ.

Supplementary Figure 5 Network of DEMs with Immune-Related Proteins and the Level of IgG, Related to Figure 6. **(A)** Correlation network of DEMs with proteins involved in the humoral immune response and regulation of humoral immune response. **(B)** Correlation network of DEMs with proteins involved in complement activation and the regulation of complement activation network. **(C)** Correlation network of DEMs with proteins involved in other immune-related network. Nodes and squares represent proteins and metabolites, respectively. Lines represent metabolite and protein associations. **(D)** Correlation analysis of IgG and DEMs, using correlations analysis. All edges are based on p<0.05.

Supplementary Figure 6 Change in Metabolites involved in Tryptophan Metabolism and Biosynthesis of Unsaturated Fatty Acids Pathways, Related to Figure 5. **(A)** Significant changes were seen in the levels of some intermediates of the tryptophan pathway in the plasma of vaccinated samples. **(B)** Changed metabolites in tryptophan pathways after vaccination. **(C)** Changed metabolites involved in the biosynthesis of unsaturated fatty acids pathway after vaccination. Changed metabolites after vaccination were labeled as red. Statistical significance was determined by paired two-sided Welch’s t test. *p < 0.05; **p < 0.01; ***p < 0.001.

Supplementary Figure 7 Plasma Cytokine Levels in Samples After Vaccination. Levels of cytokines in plasma from vaccination samples compared to baseline. Levels of IL-6 in plasma were below the detectable value. Statistical significance was determined by paired two-sided Welch’s t test. *p < 0.05; **p < 0.01; ***p < 0.001.

Supplementary Figure 8 Clinical Index in the Plasma of Vaccine Immunized Samples. **(A)** Count of white blood cells (WBC) and red blood cells (RBC). **(B)** Count of neutrophils (Neu), lymphocyte (Lym), eosinophils (Eos), monocytes (Mon) and basophils (Bas). **(C)** Proportion of Neu, Lym, Eos, Mon and Bas. **(D)** Expression of hemoglobin-related clinical indicators including hemoglobin (HGB), hematocrit (HCT), mean corpuscular volume (MCV), mean corpuscular hemoglobin (MCH), mean corpuscular hemoglobin concentration (MCHC), red blood cell distribution width-coefficient of variation (RDW-CV) and red blood cell distribution width-standard deviation (RDW-SD). **(E)** Expression of platelet-related clinical indicators including platelet (PLT), platelet volume distribution width (PDW) and plateletcrit (PCT). Significance was determined by paired two-sided Welch’s t test. *p<0.05; **p<0.01; ***p<0.001.
