# Supplementary figures and images for: Proteomic and Metabolomic Signatures Associated With the Immune Response in Healthy Individuals Immunized With an Inactivated SARS-CoV-2 Vaccine
Source: Front Immunol. 2022 May 24;13:848961. doi: 10.3389/fimmu.2022.848961 (PMC9171821; doi:10.3389/fimmu.2022.848961)

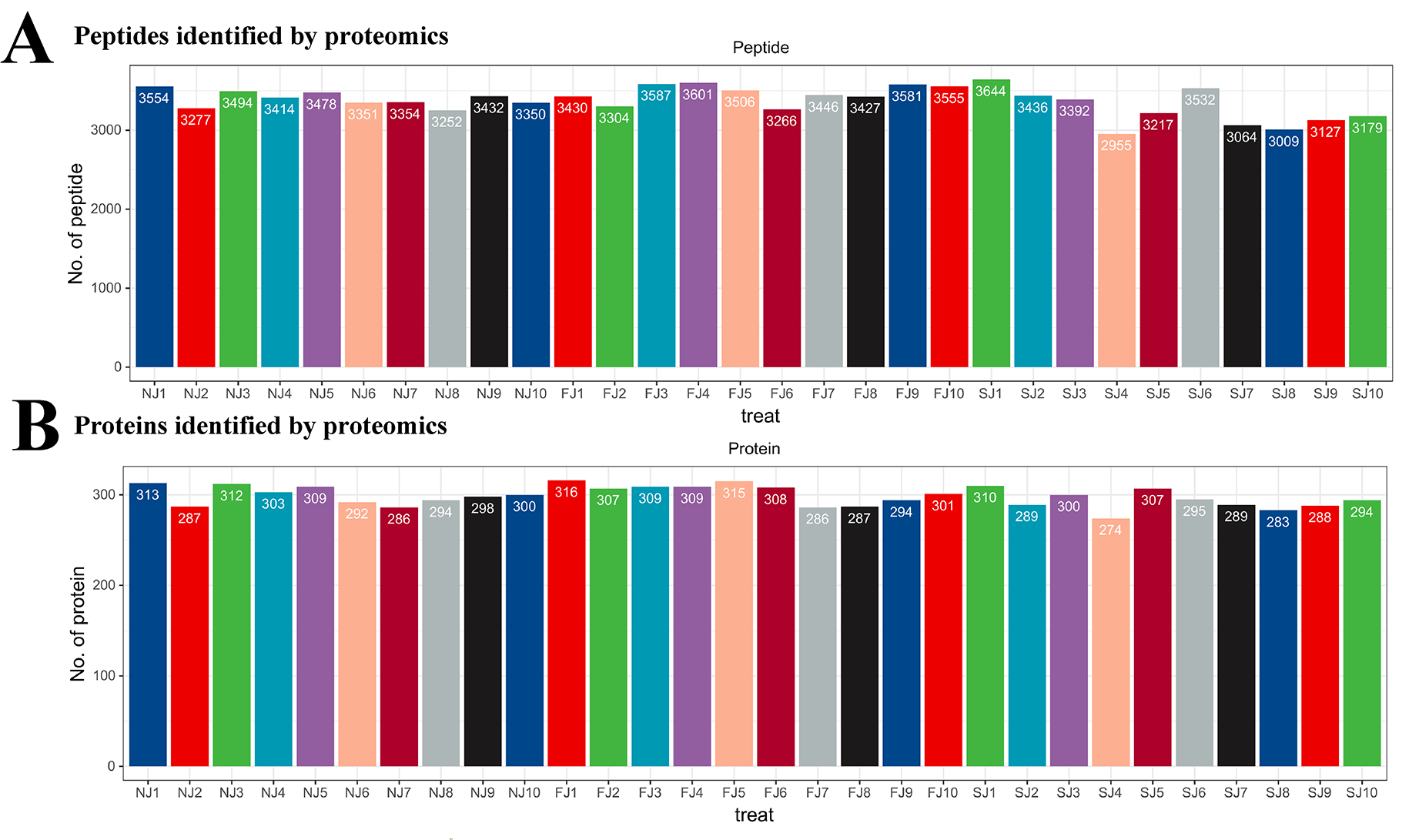

Supplement: Supplementary Figure 1 — Quality Control of Proteomic and Metabolomic Data. (A) The distribution of the numbers of quantified peptides in the 30 plasma samples. (B) The distribution of the numbers of quantified proteins in the 30 plasma samples. [file Image_1.tif]

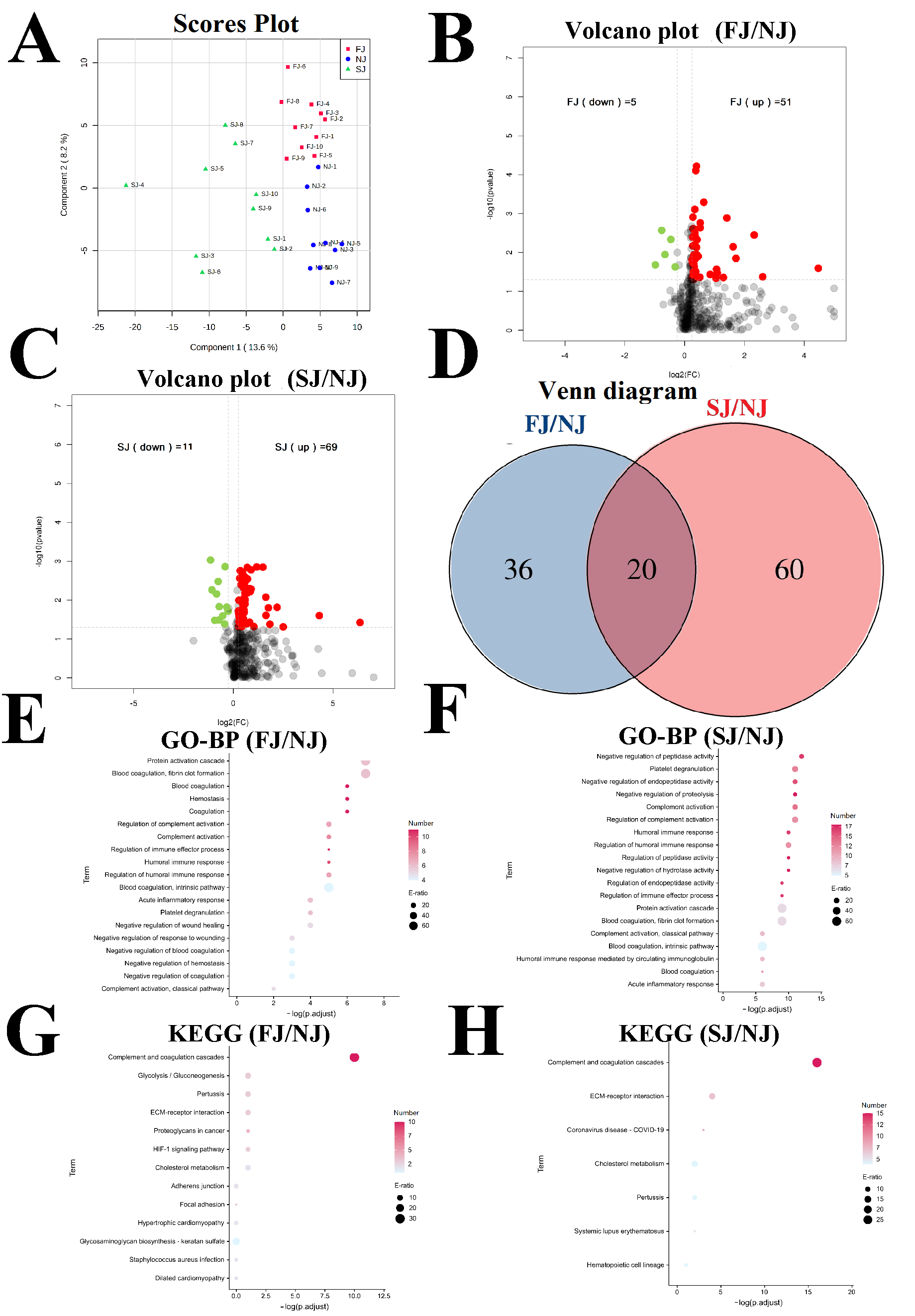

Supplement: Supplementary Figure 2 — Differentially Expressed Proteins in Different Groups, Related to Figure 2. (A) Partial Least Squares Discriminant Analysis (PLS-DA) was used to compare the proteins of the different groups. (B) Volcano plot comparing protein expression in FJ vs NJ. (C) Volcano plot comparing protein expression in SJ vs NJ. Proteins with log2 (fold-change) above 0.25 or below -0.25 and p value <0.05 were considered to be significantly DEPs. Number of significantly down- (green) and up- (red) regulated proteins are shown on top. (D) Venn diagram of the number of DEPs. (E) GO-BP analysis of the DEPs from FJ vs NJ. (F) KEGG pathway analysis of the DEPs from FJ vs NJ. (G) GO-BP analysis of the DEPs from SJ vs NJ. (H) KEGG pathway analysis of the DEPs from FJ vs NJ. Top GO-BP and KEGG terms were expressed. [file Image_2.tif]

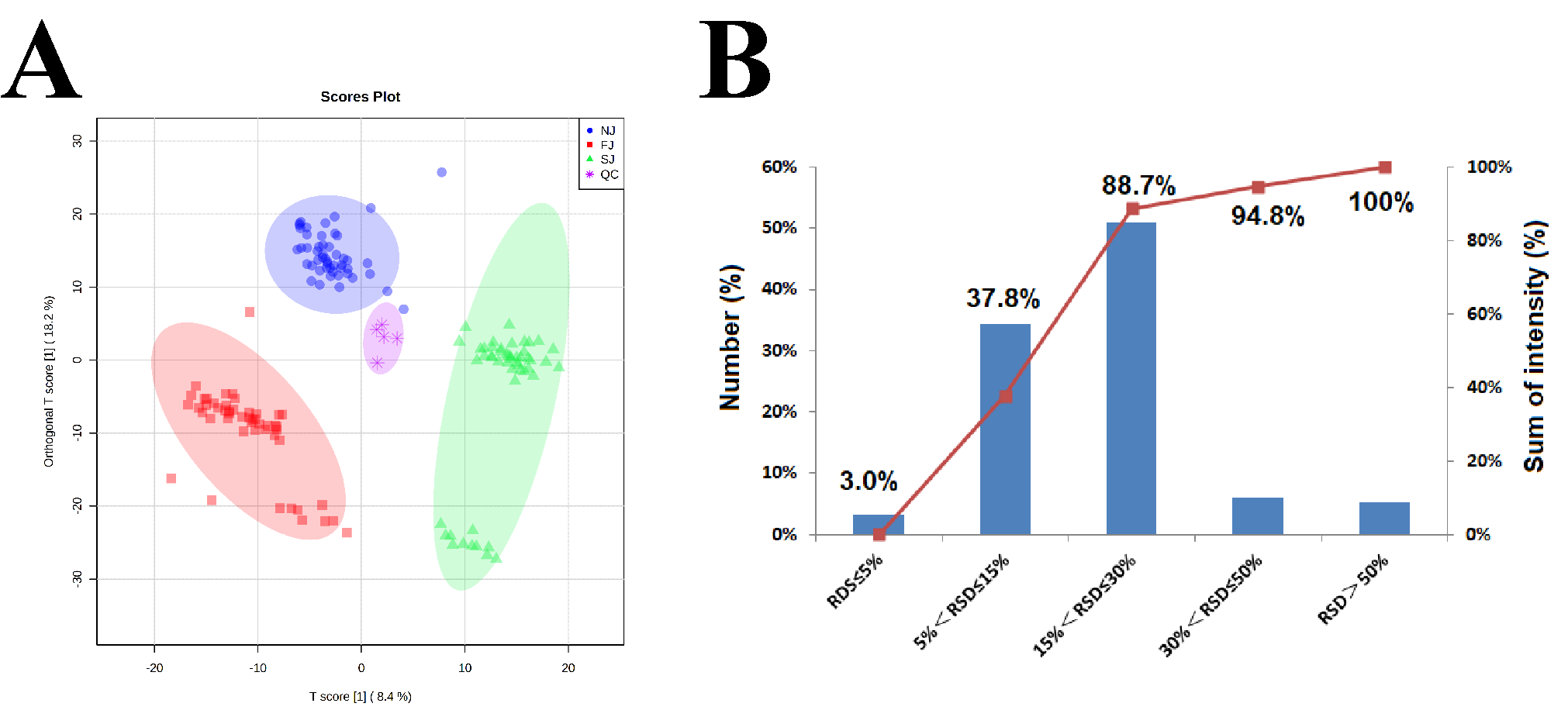

Supplement: Supplementary Figure 3 — Quality Control of Metabolomics. (A) Reliability investigation of the analytical method using QC samples (n = 5). Score plot of Orthogonal Partial Least Squares Discrimination Analysis (OPLS-DA). (B) RSD% of all detected variables. Number (%): percentage of number of variables. Sum of intensity (%): percentage of total response of variables. [file Image_3.tif]

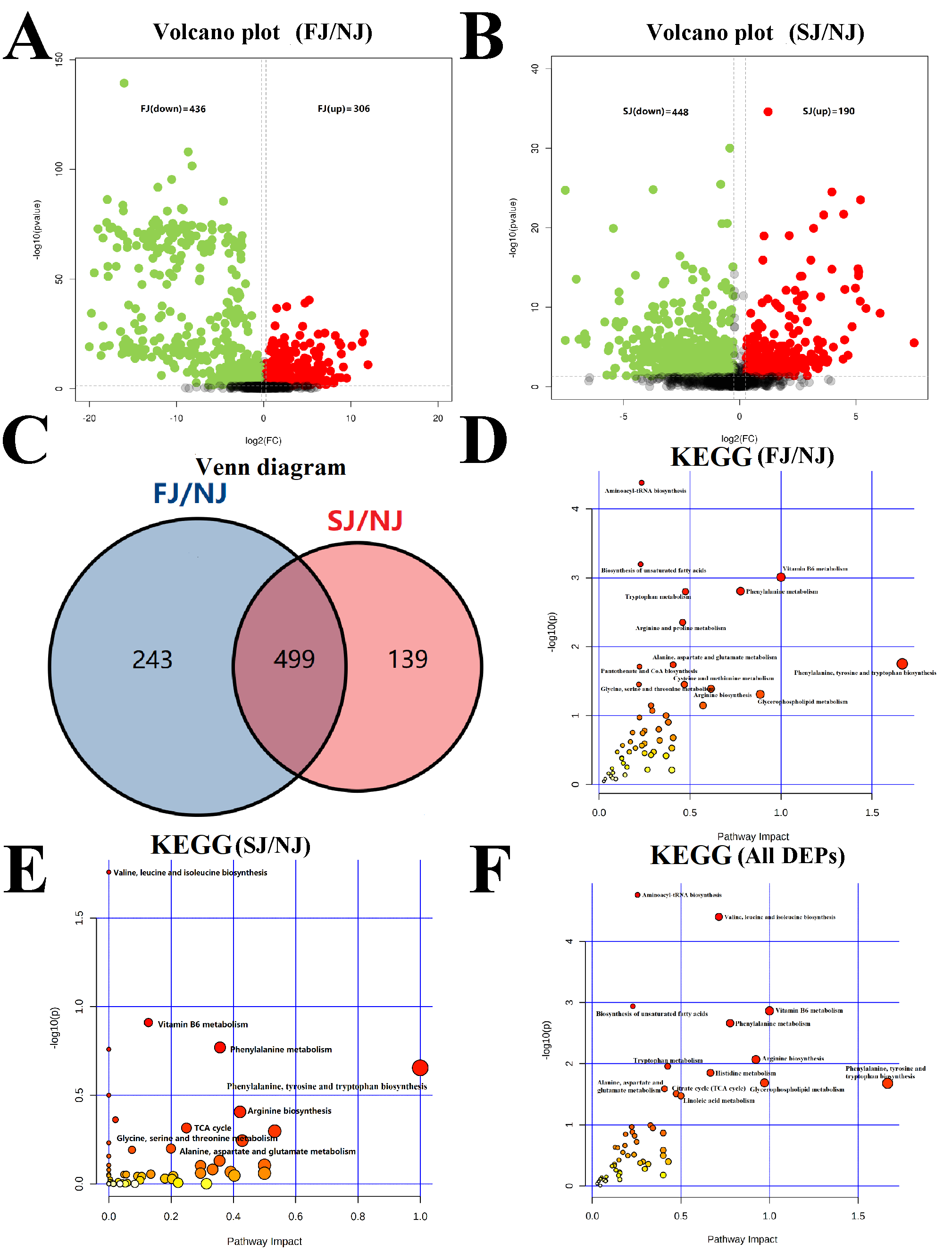

Supplement: Supplementary Figure 4 — Differentially Expressed Metabolites in Different Groups, Related to Figure 4. (A) Volcano plot comparing metabolite expression in FJ vs NJ. (B) Volcano plot comparing metabolite expression in SJ vs NJ. Metabolites with log2 (fold-change) above 0.25 or below 0.25 with p value <0.05 were considered to be DEMs. Number of significantly down- (green) and up- (red) regulated proteins are shown on top. (C) Venn diagram showing the number of DEMs. (D) KEGG pathway analysis of the DEMs from FJ vs NJ. (D) KEGG pathway analysis of the DEMs from SJ vs NJ. (E) KEGG pathway analysis of the total DEMs from FJ vs NJ and SJ vs NJ. [file Image_4.tif]

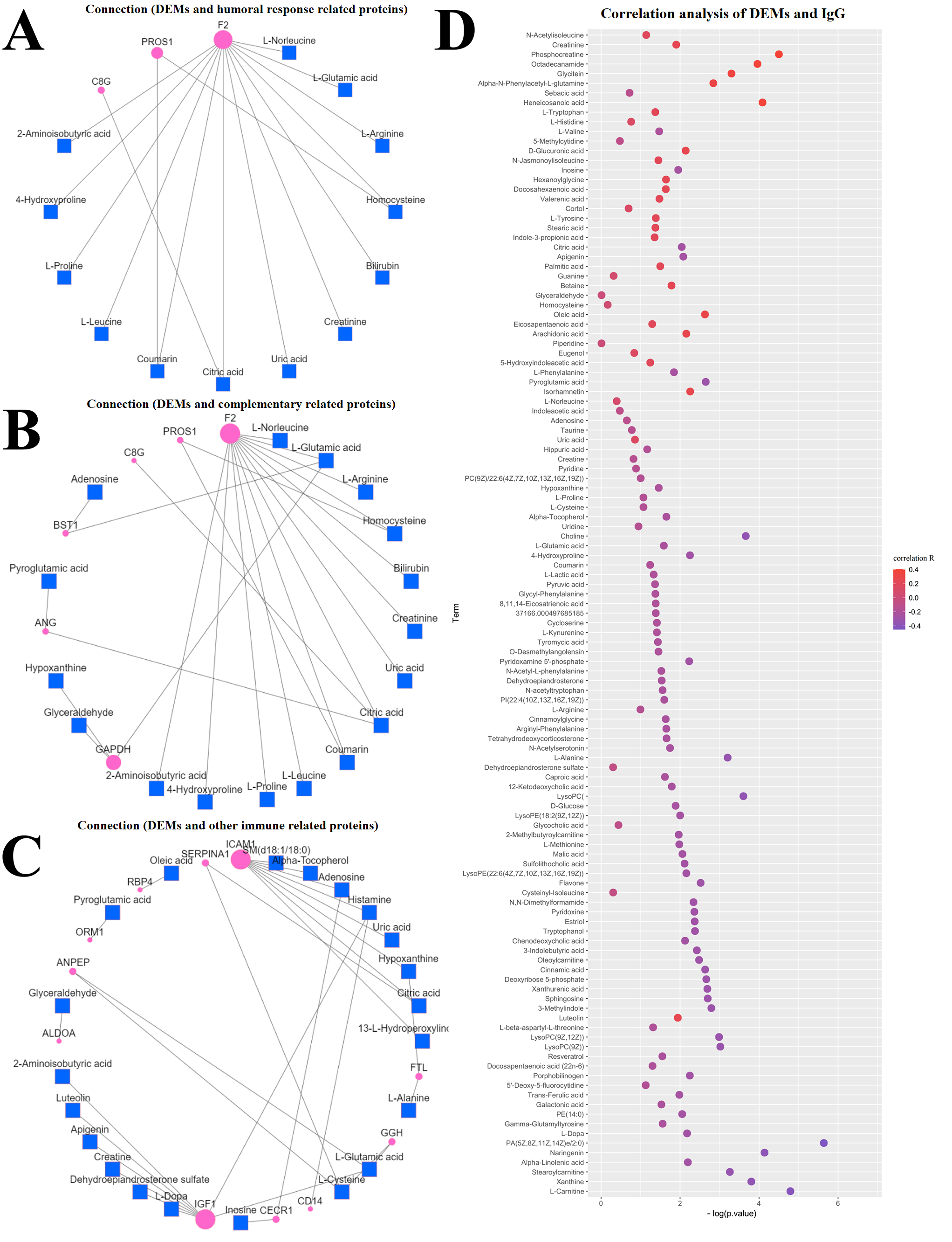

Supplement: Supplementary Figure 5 — Network of DEMs with Immune-Related Proteins and the Level of IgG, Related to Figure 6. (A) Correlation network of DEMs with proteins involved in the humoral immune response and regulation of humoral immune response. (B) Correlation network of DEMs with proteins involved in complement activation and the regulation of complement activation network. (C) Correlation network of DEMs with proteins involved in other immune-related network. Nodes and squares represent proteins and metabolites, respectively. Lines represent metabolite and protein associations. (D) Correlation analysis of IgG and DEMs, using correlations analysis. All edges are based on p<0.05. [file Image_5.tif]

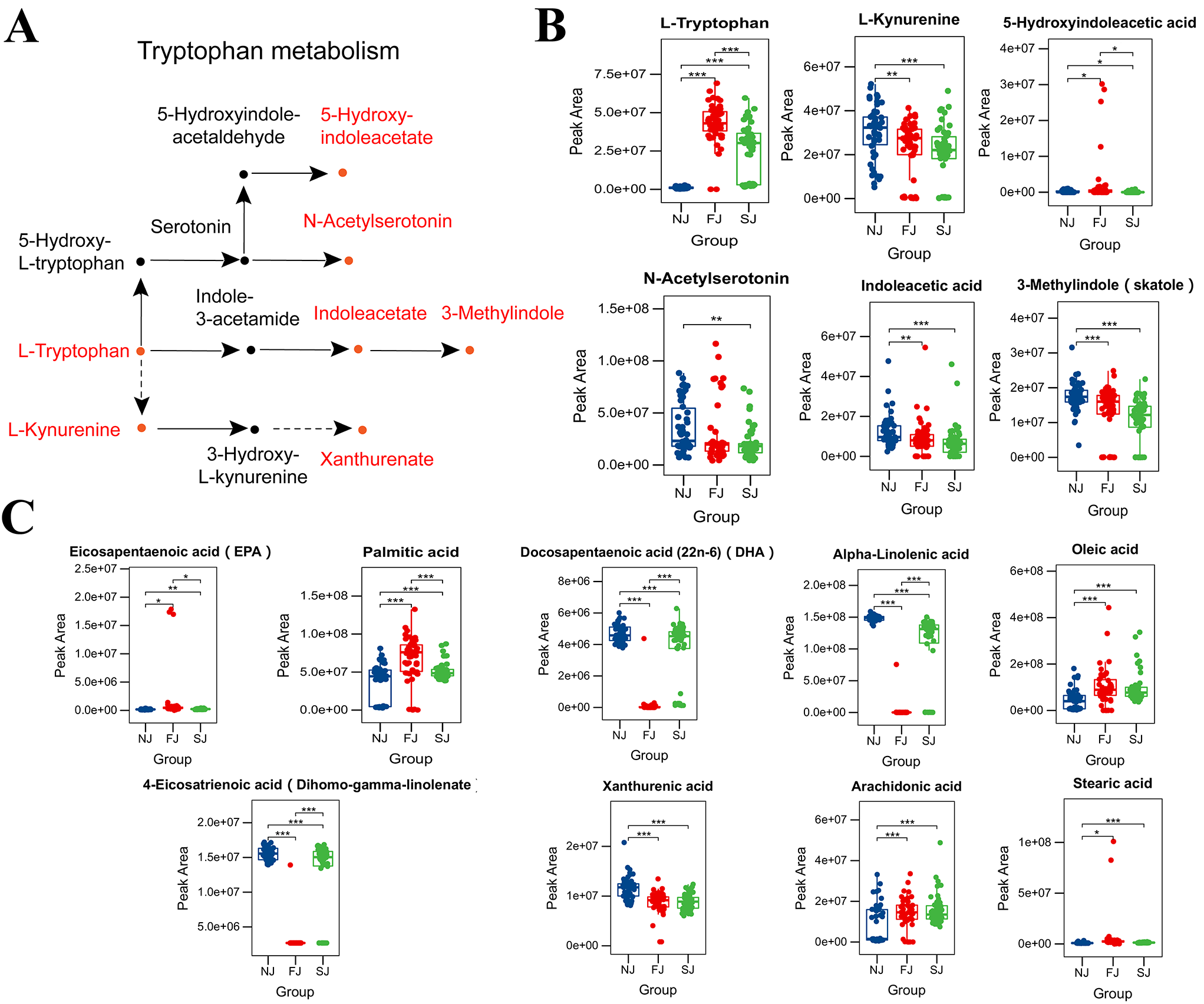

Supplement: Supplementary Figure 6 — Change in Metabolites involved in Tryptophan Metabolism and Biosynthesis of Unsaturated Fatty Acids Pathways, Related to Figure 5. (A) Significant changes were seen in the levels of some intermediates of the tryptophan pathway in the plasma of vaccinated samples. (B) Changed metabolites in tryptophan pathways after vaccination. (C) Changed metabolites involved in the biosynthesis of unsaturated fatty acids pathway after vaccination. Changed metabolites after vaccination were labeled as red. Statistical significance was determined by paired two-sided Welch’s t test. *p < 0.05; **p < 0.01; ***p < 0.001. [file Image_6.tif]

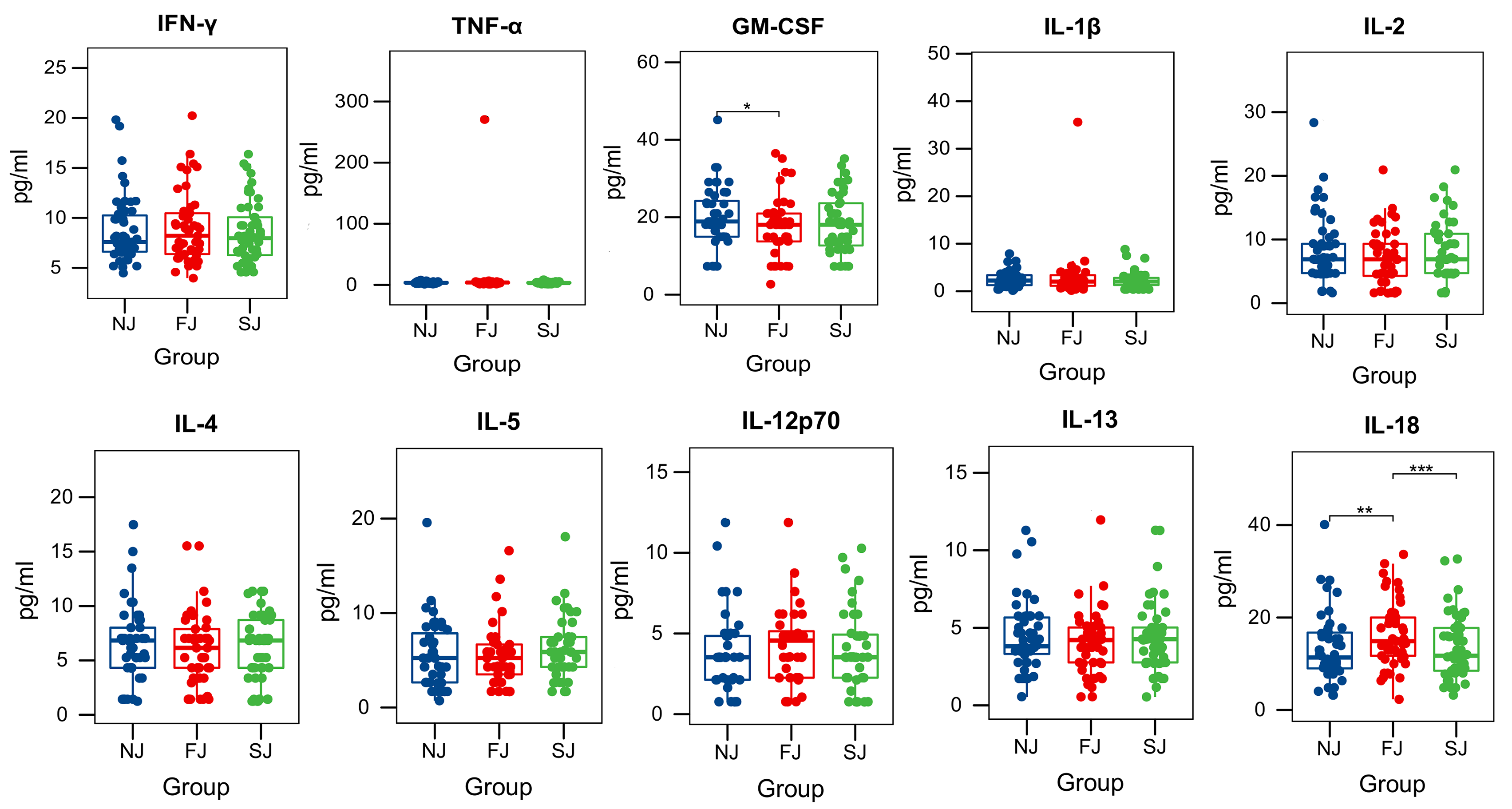

Supplement: Supplementary Figure 7 — Plasma Cytokine Levels in Samples After Vaccination. Levels of cytokines in plasma from vaccination samples compared to baseline. Levels of IL-6 in plasma were below the detectable value. Statistical significance was determined by paired two-sided Welch’s t test. *p < 0.05; **p < 0.01; ***p < 0.001. [file Image_7.tif]

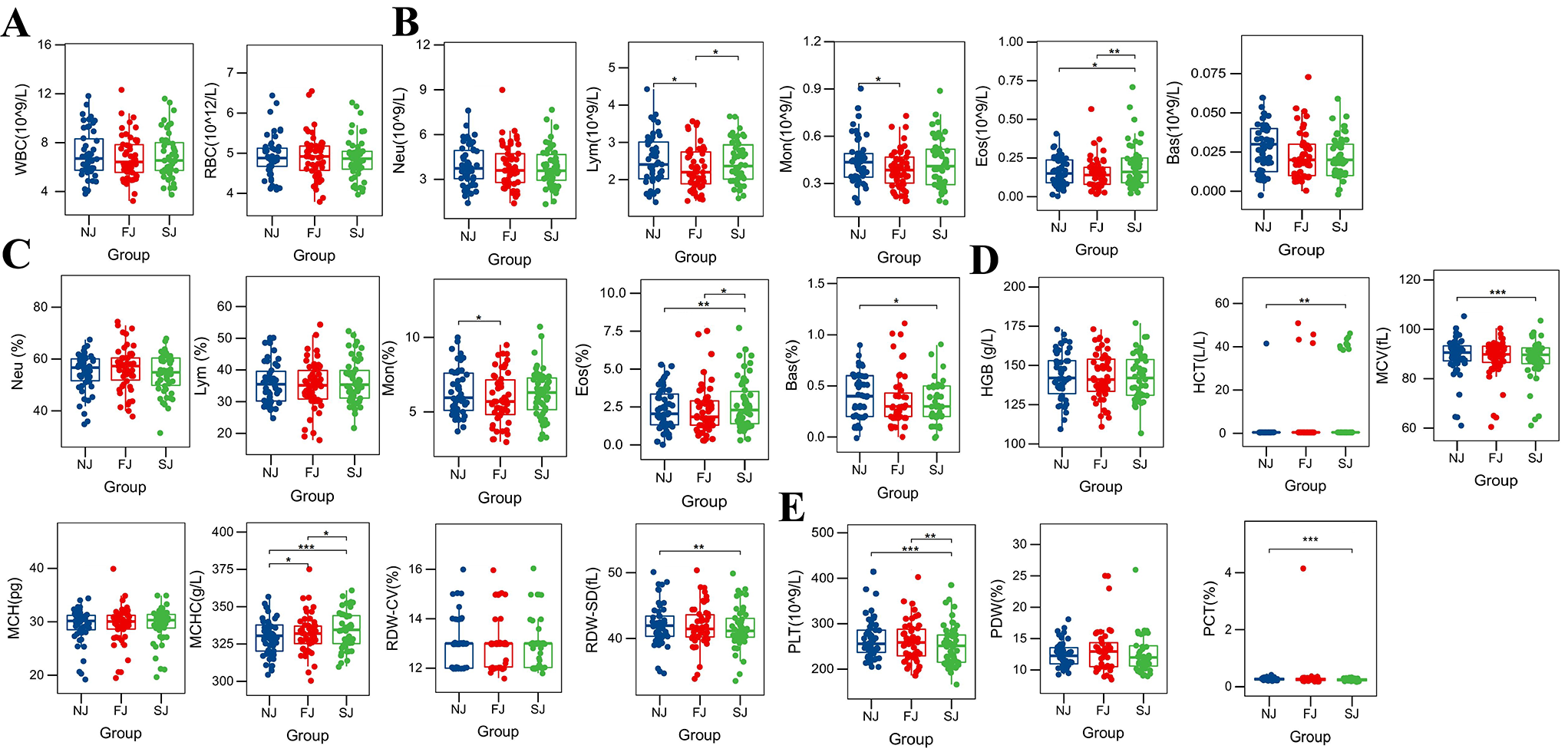

Supplement: Supplementary Figure 8 — Clinical Index in the Plasma of Vaccine Immunized Samples. (A) Count of white blood cells (WBC) and red blood cells (RBC). (B) Count of neutrophils (Neu), lymphocyte (Lym), eosinophils (Eos), monocytes (Mon) and basophils (Bas). (C) Proportion of Neu, Lym, Eos, Mon and Bas. (D) Expression of hemoglobin-related clinical indicators including hemoglobin (HGB), hematocrit (HCT), mean corpuscular volume (MCV), mean corpuscular hemoglobin (MCH), mean corpuscular hemoglobin concentration (MCHC), red blood cell distribution width-coefficient of variation (RDW-CV) and red blood cell distribution width-standard deviation (RDW-SD). (E) Expression of platelet-related clinical indicators including platelet (PLT), platelet volume distribution width (PDW) and plateletcrit (PCT). Significance was determined by paired two-sided Welch’s t test. *p<0.05; **p<0.01; ***p<0.001. [file Image_8.tif]
